# Long-Term Impact of Parental Post-Traumatic Stress Disorder Symptoms on Mental Health of Their Offspring After the Great East Japan Earthquake

**DOI:** 10.3389/fpsyt.2019.00496

**Published:** 2019-07-26

**Authors:** Yukiko Honda, Takeo Fujiwara, Junko Yagi, Hiroaki Homma, Hirobumi Mashiko, Keizo Nagao, Makiko Okuyama, Masako Ono-Kihara, Masahiro Kihara

**Affiliations:** ^1^Division of Advanced Preventive Medical Sciences, Graduate School of Biomedical Sciences, Nagasaki University, Nagasaki, Japan; ^2^Faculty of Advanced Science and Technology, Kumamoto University, Kumamoto, Japan; ^3^Department of Global Health and Socio-epidemiology, School of Public Health, Graduate School of Medicine, Kyoto University, Kyoto, Japan; ^4^Department of Social Medicine, National Research Institute for Child Health and Development, Tokyo, Japan; ^5^Department of Global Health Promotion, Tokyo Medical and Dental University, Tokyo, Japan; ^6^Department of Psychiatry, Iwate Medical University, Iwate, Japan; ^7^Asaka Hospital, Fukushima, Japan; ^8^Fukushima Rehabilitation Center for Children, Fukushima, Japan; ^9^Nagao Mental Clinic, Mie, Japan; ^10^Department of Psychosocial Medicine, National Center for Child Health and Development, Tokyo, Japan

**Keywords:** disaster, behavior problems, mental health, post-traumatic stress disorder, long-term impact, parent-child interaction, Japan

## Abstract

Longitudinal studies of the long-term psychological impact of the Great East Japan Earthquake (GEJE) on parents and their children have been limited. The current study aimed to monitor parents’ post-traumatic stress disorder (PTSD) symptoms and behavioral problems among their children over time and to analyze their long-term associations among the survivors of the GEJE. We used data from the GEJE Follow-up for Children study, which recruited 4- to 6-year-old children and those children’s parents immediately after the GEJE in March 2011, with ongoing follow-up. Children’s total, internalizing, and externalizing behavioral problems were assessed using the Child Behavior Checklist (CBCL), and parental probable PTSD was assessed using the Impact of Event Scale-R (IES-R), in 2012 (baseline) and 2014 (follow-up). Parental PTSD symptoms and children’s behavioral problems declined slightly over time, and both showed a significant correlation between the surveys (r = 0.55–0.77, *P* < 0.001). The association between parental PTSD symptoms and children’s behavioral problems was investigated using multivariate logistic regression analysis adjusting for baseline children’s behavioral problems and other potential confounders. Cross-sectionally, while no significant association was detected in 2012, all types of children’s behavioral problems exhibited significant positive associations with parental PTSD symptoms in multiple logistic regression analysis adjusted odds ratio (AOR) = 3.03, 3.30, and 5.34 for total, internalizing, and externalizing behavior problems, respectively. Maternal educational attainment level (higher than high school education) showed a significant negative association with children’s total and externalizing behavioral problems (AOR = 0.30 and 0.13, respectively) in 2014. Longitudinally, parental PTSD symptoms in 2012 showed a significant association with children’s internalizing behavioral problems in 2014 after adjusting for children’s behavioral problems in 2012 and parental PTSD symptoms in 2014 (AOR = 4.62). These results suggest that the effect of the GEJE on parental PTSD symptoms and children’s behavioral problems was long-term, lasting for at least 3 years. These possibilities should be carefully considered in mental health support for parents and their offspring in areas affected by the GEJE.

## Introduction

Natural disasters often constitute severe crises, involving damage to children’s mental health directly or indirectly, causing mental and behavioral problems. There is a growing body of research examining children’s psychological responses to traumatic events, including post-traumatic stress disorder (PTSD), depression, and behavioral problems (1–6). The prevalence rate of PTSD symptoms among children in Thailand 2 months after the 2004 Indian Ocean earthquake and tsunami was 6% to 11%, and the rate of depression was 5% to 11% (1). Following the Sichuan earthquake in 2008 in China, 11% to 28% of children suffered from PTSD symptoms (7, 8), 14% to 19% suffered from depression (7, 8), and 22% to 23% suffered from anxiety (7). Further, the Great East Japan Earthquake (GEJE) in 2011 caused behavioral problems among 26% of young children (9).

Several risk factors have been suggested for behavioral problems among children after natural disasters, including traumatic experience, poor living conditions, low socioeconomic status (SES), parental education, and parental mental health (5, 6, 9–15). Among these risk factors, parental mental health has been reported to play an important role in the behavioral problems of offspring (16, 17). Scheeringa et al. reviewed the association between parental trauma-related mental health illness, including PTSD symptoms, and mental health problems among children (16, 17) in cases of natural disasters (e.g., blizzard, hurricane, or brush fire), human-made disasters (terrorism, nuclear power plant explosions, or war), or interpersonal trauma (e.g., rape, child maltreatment, interpersonal violence) in the US, Croatia, and Cambodia, among children at a range of ages, from toddlers to adolescents. The results revealed a robust association between parental mental health and behavioral problems among offspring, suggesting that parental psychopathology may have a causal effect on children’s behavioral problems after traumatic exposure (16, 17). However, because most previous studies have used cross-sectional study designs (11, 18–26–28), the potential long-term impact of parental PTSD on children’s mental health remains unclear. To the best of our knowledge, three studies, one on bushfire and the other two on human-made disasters, reported an association using a longitudinal design (29–31). However, these studies are relatively old, and require replication in other settings, such as Japan, which experiences earthquakes frequently (e.g., the Hanshin-Awaji Great Earthquake in 1995, the GEJE in 2011, and the Kumamoto Earthquake in 2016).

The GEJE Follow-up for Children (GEJE-FC) study is an ongoing longitudinal cohort study started in 2012, collecting data about children and their parents who were negatively impacted by the GEJE. In this cohort study, parental PTSD symptoms and behavioral problems of their offspring have been repeatedly assessed, enabling us to evaluate the impact of parental PTSD symptoms on behavioral problems among their offspring using a longitudinal design. The aim of the current study was to evaluate parental PTSD symptoms, the behavioral problems of their offspring, and the association between them, on a long-term basis, following the GEJE.

## Materials and Methods

### Participants

We used data from the GEJE-FC study; details of the sampling strategy have been described elsewhere (9). The children originally targeted were 4–6 years old, and were attending nursery schools or kindergarten at the time of the earthquake in March 2011. In the GEJE-FC study, child participants were required for being able to memorize earthquakes and express their feelings to their parents. Therefore, the children in 4 to 6 years old were targeted, considering their developmental stage for the skills above. The parents of those children were also surveyed in conjunction with their children. At the baseline survey from August 2012 to June 2013, we recruited participants in multiple stages. First, we selected the municipalities impacted by the GEJE in three prefectures (Iwate, Miyagi, and Fukushima) as affected regions, and selected those in an unaffected prefecture (Mie) as a comparison region. Second, we selected nursery schools in each municipality of each prefecture. Finally, we asked the staff of the selected nursery schools to recruit parents to participate in the study together with their children. As we only targeted participants who were victim, living in the region of the earthquake at the time of the event in the current study, we excluded data from the comparison region.

**Figure 1:**
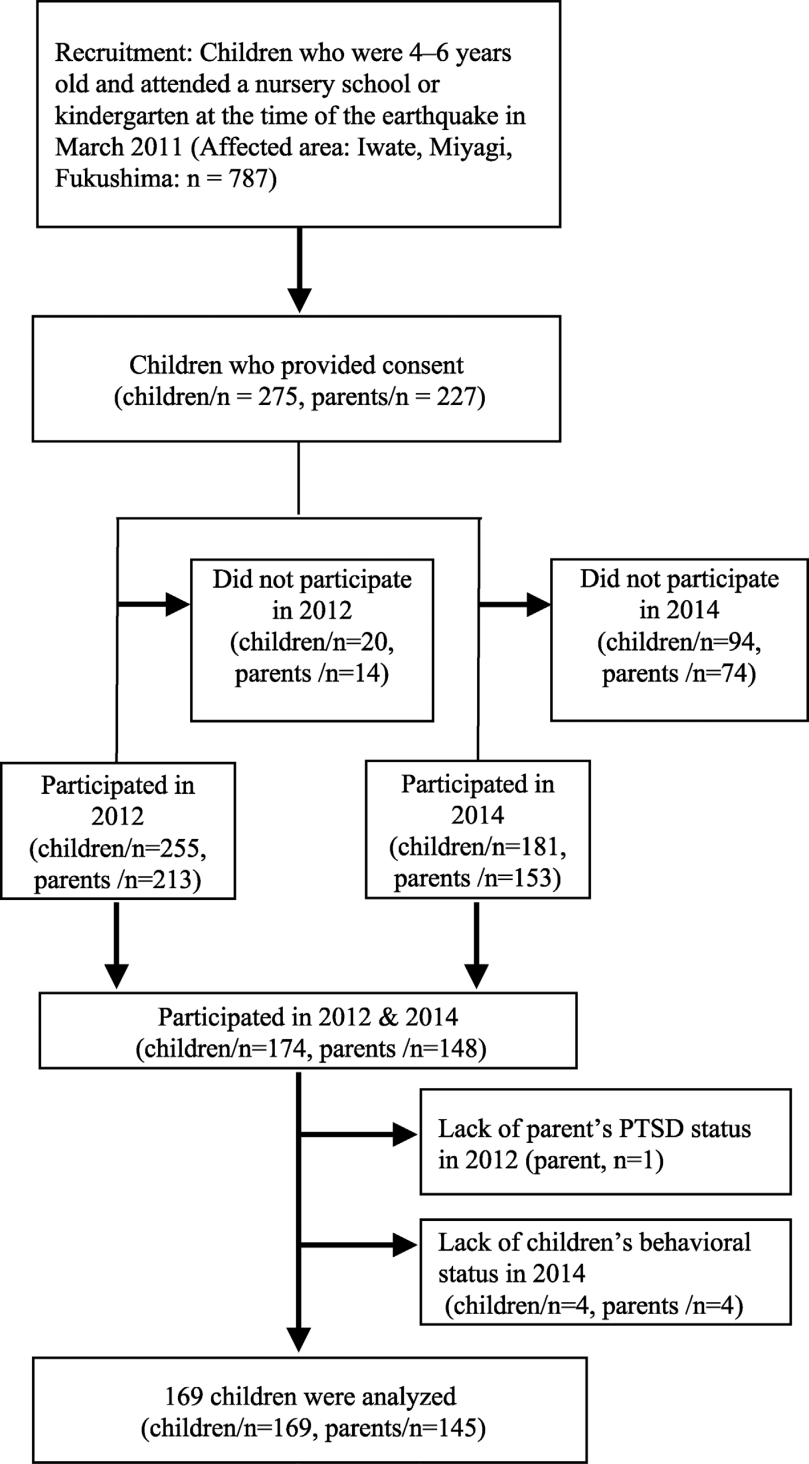
Flowchart of the sample selection process.

A total of 275 children and 227 of their parents initially agreed to participate. Written informed consent was obtained from parents and their children by the research coordinators, after providing oral and written explanations of the study. The numbers of children and parents who actually participated in the survey were 255 and 213, respectively, in 2012 (baseline), and 181 and 153, respectively, in 2014 (follow-up, from July to December 2014). In the current study, we included children (n = 174) and their parents (n = 148) who participated in the survey in both years (2012 and 2014), and excluded records that lacked information about child behavioral problems in 2014 (n = 4) or parental PTSD symptoms in 2012 (n = 1). Finally, data from 169 children and 145 of their parents were analyzed in this study (**Figure 1**). Of 169 children, 24 were siblings with the same parents. The original GEJE-FC study was approved by the ethics committees at the National Center for Child Health and Development and Tokyo Medical and Dental University. Ethical approval was also sought for this secondary data analysis from the Kyoto University Graduate School and Faculty of Medicine Ethics Committee.

### Measurements

#### PTSD Symptoms Among Parents

We assessed PTSD symptoms of parents using the Impact of Event Scale-Revised (IES-R) (32), which has been assessed for validity and reliability in four studies of different traumatic events in Japan (33). Scores ranged 0–88, and the cutoff score for having probable PTSD was set at 24 or more, based on a previous study in Japan (33).

### Children’s Behavioral Problems

We assessed children’s behavioral problems using the Child Behavior Checklist (CBCL), which was administered by caregivers (34, 35). The CBCL classifies children’s behavioral problems into three categories: internalizing behavioral problems (withdrawn behavior, somatic complaints, anxiety or depression), externalizing behavioral problems (rule-breaking or aggressive behavior) and total behavioral problems (social problems, thought problems and attention problems are added to the internalizing and externalizing behavioral problems). The T score (mean = 50, standard deviation = 10) of CBCL scores was calculated and standardized for Japanese children (35), for both 2012 and 2014.

### Children’s Exposure to Disaster-Related Trauma

We assessed children’s exposure to disaster-related traumatic experiences through interviews by psychiatrists or clinical psychologists, including separation from the parents, death of close family member(s), death of other close persons such as relatives or friends, watching tsunami waves or victims being swept away by the tsunami, exposure to fire, or witnessing a dead body. We also assessed other traumatic events including the loss of family, damage to the house (wholly or partially), evacuating a shelter, living in a temporary house, or separation from the family, using a questionnaire. We combined these data to calculate the total number of traumatic experiences of each child.

### Statistical Analysis

Statistical analysis was conducted in three steps. First, the cross-sectional associations between parental PTSD symptoms and children’s behavioral problems were evaluated in each survey using bivariate and multivariate logistic regression analysis, adjusting for child factors (age, sex, traumatic experiences), parental factors (age, education level), family factors (number of siblings and household income), and further adjusting for familial clustering of the children. Second, we assessed Pearson’s correlations between the 2012 and 2014 scores for parental PTSD symptoms and children’s behavioral problems, respectively. Finally, to assess the long-term impact of parental PTSD on children’s behavioral problems, the association between parental PTSD symptoms in 2012 and children’s behavioral problems in 2014 was analyzed using bivariate and multivariate logistic regression analyses, adjusting for children’s behavioral problems in 2012, parental PTSD symptoms in 2014, and other covariates. We used Stata MP version 14.2 (STATA Corp., College Station, TX) for analysis.

## Results

### Characteristics of the Participants

The demographic characteristics and mental health status of participants are shown in **Table 1**. For children, the mean age was 6.7 years (SD = 1.4) with almost equal gender distribution in 2012 (baseline). T scores for behavioral problems measured by CBCL exceeded 50 (mean of Japanese children) in both 2012 and 2014 for total, internalizing, and externalizing behavioral problems with a tendency of scores in 2014 being consistently smaller than those in 2012. Although it was not shown in the table, children who were assessed as within the clinical range (cutoff score > 63 (35) in total behavioral problems were 18.9% at baseline and 20.1% in the follow-up. More than 80% of the children experienced one or more traumatic experiences. After GEJE, some children had consulted with the medical doctors, clinical psychologists, or educator on their mental health or behavior problems (19.5%), and also had visited the clinics or hospitals to receive medical care on those problems (10.1%). For parents, mean age was 36.8 years (SD, 6.0) at baseline, with most of them being mothers (93.1%). Although the other parents were fathers (4.1%), grandfather (0.7%), and missing value (2.1%), almost all of the “parents” were mothers in the current study. Half of the parents (49.0%) reported that their highest level of educational attainment was high school or less. The prevalence of probable PTSD (score ≥ 24) among parents was 24.1% in 2012 and 19.3% in 2014 with a tendency to reduce over time. In terms of family size, 75.2% of children had one or more siblings, while 22.5% had no siblings. More than one-fifth of families (21.9%) reported that their household income was less than JPY 3,000,000, substantially lower than the average annual income in Japan (JPY 4,150,000, approx. USD 4 million) (36) at the survey in 2014.

**Table 1:** Demographic and familial characteristics of participants at baseline (2012) and children’s behavioral and maternal mental health problems at both baseline and follow-up (2014).

		Total (N = 169)	
Characteristics		Mean or N	SD or (%)
Children			
Age in 2012 (years)		6.7	1.4
Sex			
	Boy	88	(52.1)
	Girl	81	(47.9)
CBCL behavior problems (2012, n = 149)			
	Total (T score)	56.1	10.6
	Internalizing (T score)	55.9	9.1
	Externalizing (T score)	55.7	10.0
CBCL behavior problems (2014, n = 169)			
	Total (T score)	53.1	11.0
	Internalizing (T score)	53.9	9.3
	Externalizing (T score)	52.8	9.9
Number of traumatic experiences (2012)			
	0	33	(19.5)
	1-2	49	(29.0)
	3-4	54	(32.0)
	5+	33	(19.5)
Parents			
Responder (2012)			
	Mother	135	(93.1)
	Father	6	(4.1)
	Grand father	1	(0.7)
	Missing	3	(2.1)
Age in 2012 (years)		36.8	6.0
Education (2012)			
	High school or less	71	(49.0)
	Some college+	68	(46.9)
	Missing	6	(4.1)
PTSD symptoms (2012, n = 145)			
	IES-R total score	16.4	14.8
	IES-R total score >24	35	(24.1)
PTSD symptoms (2014, n = 145)			
	IES-R total score	14.0	13.6
	IES-R total score >24	28	(19.3)
Familial			
Number of siblings (2012)			
	0	38	(22.5)
	1	77	(45.6)
	2+	50	(29.6)
	Missing	4	(2.4)
Annual household income (2014)			
(Unit: JPY10,000)	<300	37	(21.9)
	300-599	67	(39.6)
	600+	43	(25.4)
	Missing	22	(13.0)

CBCL, The child behavior checklist; PTSD, post-traumatic stress disorder; IES-R, the impact of event scale-revised; SD, standard deviation.

### Correlation Between Scores in 2012 (Baseline) and 2014 (Follow-Up) for Parental PTSD Symptoms and Children’s Behavioral Problems

**Table 2** summarizes the correlations between scores in 2012 (baseline) and 2014 (follow-up) for parental probable PTSD symptoms and children’s behavioral problems. All types of children’s behavioral problems showed a strong correlation (*r* = 0.64–0.77, p < 0.001), while parental PTSD symptoms showed a moderate correlation (*r* = 0.55, p < 0.001) between two surveys.

**Table 2:** Correlations between scores in 2012 (baseline) and 2014 (follow-up) for parental PTSD symptoms and children’s behavioral problems.

Variables	r	95% CI	p value
Parental PTSD symptoms	0.55	0.44–0.65	<0.001
Children’s total behavior problems	0.77	0.70–0.83	<0.001
Children’s internalizing behavior problems	0.64	0.53–0.72	<0.001
Children’s externalizing behavior problems	0.74	0.66–0.81	<0.001

PTSD, post-traumatic stress disorder; r, Pearson’s correlation coefficient; CI, confidence interval.

### Cross-Sectional Association Between Parental PTSD Symptoms and Children’s Behavioral Problems in 2012 (Baseline) and 2014 (Follow-Up)

**Table 3** shows the cross-sectional relationship between parental PTSD symptoms and children’s behavioral problems in 2012 (baseline). The results revealed no significant associations between children’s behavioral problems and any of the covariates in the multivariate model.

**Table 3:** Association between parental PTSD symptoms and children’s behavioral problems in 2012.

		Total behavior problems				Internalizing behavior problems				Externalizing behavior problems			
		Bivariate		Multivariate (n = 211)		Bivariate		Multivariate (n = 211)		Bivariate		Multivariate (n = 211)	
		OR	p	AOR	p	OR	p	AOR	p	OR	p	AOR	p
		(95%CI)		(95%CI)		(95%CI)		(95%CI)		(95%CI)		(95%CI)	
Parental PTSD Symptoms													
	Non clinical	ref.		ref.		ref.		ref.		ref.		ref.	
	Clinical	1.75	0.13	1.78	0.23	1.67	0.16	1.58	0.32	**2.06**	**0.05**	2.11	0.11
		(0.85–3.58)		(0.70–4.49)		(0.82–3.41)		(0.65–3.82)		**(0.99–4.30)**		(0.86–5.20)	
Age of child (years)		a				a				a			
		1.29	0.09	1.18	0.30	1.22	0.16	1.25	0.14	1.13	0.43	1.05	0.75
		(0.96–1.74)		(0.86–1.60)		(0.93–1.61)		(0.93–1.70)		(0.84–1.52)		(0.77–1.44)	
Sex of child													
	Boy	ref.		ref.		ref.		ref.		ref.		ref.	
	Girl	0.64	0.17	0.65	0.24	0.59	0.11	0.52	0.09	0.86	0.64	0.79	0.50
		(0.34–1.20)		(0.32-1.33)		(0.31–1.13)		(0.25–1.11)		(0.45–1.63)		(0.39–1.57)	
Number of siblings		b								b			
	0	ref.		ref.		ref.		ref.		ref.		ref.	
	1	0.67	0.30	0.56	0.18	1.32	0.49	1.20	0.69	0.78	0.55	0.68	0.40
		(0.31–1.43)		(0.24–1.31)		(0.60–2.92)		(0.49–2.91)		(0.34–1.76)		(0.28–1.66)	
	2+	0.42	0.09	0.42	0.12	1.04	0.94	1.38	0.52	0.94	0.89	0.79	0.65
		(0.16–1.15)		(0.14–1.24)		(0.41–2.65)		(0.51–3.74)		(0.37–2.34)		(0.30–2.12)	
	Missing	1.00		1.00		3.42	0.40	1.00		1.00		1.00	
		(-)		(-)		(0.20–59.25)		(-)		(-)		(-)	
Traumatic experiences													
(number)	0	ref.		ref.		ref.		ref.		ref.		ref.	
	1-2	1.67	0.30	1.32	0.61	0.95	0.91	0.71	0.49	1.61	0.31	1.34	0.54
		(0.64–4.39)		(0.44–3.99)		(0.40–2.24)		(0.26–1.89)		(0.64–4.03)		(0.53–3.43)	
	3-4	1.45	0.49	1.20	0.76	0.77	0.59	0.60	0.34	1.00	1.00	0.96	0.94
		(0.50–4.19)		(0.36–3.97)		(0.29–2.01)		(0.20–1.74)		(0.35–2.86)		(0.31–2.96)	
	5+	**3.30**	**0.03**	2.84	0.13	1.56	0.38	1.40	0.54	1.52	0.44	1.08	0.90
		**(1.10–9.93)**		(0.75–10.80)		(0.58–4.20)		(0.48–4.11)		(0.53–4.42)		(0.31–3.75)	
Age of parent (years)													
		1.00	0.96	1.00	0.88	0.97	0.18	0.95	0.07	1.02	0.34	1.05	0.11
		(0.95–1.05)		(0.95–1.06)		(0.93–1.01)		(0.89–1.00)		(0.98–1.07)		(0.99–1.10)	
Parental education		c								c			
	High school or less	ref.		ref.		ref.		ref.		ref.		ref.	
	Some college+	1.05	0.89	1.04	0.93	1.48	0.24	1.72	0.17	0.76	0.44	0.69	0.35
		(0.53–2.06)		(0.47––2.27)		(0.77–2.85)		(0.80–3.70)		(0.38–1.53)		(0.32–1.50)	
	Missing	1.00		1.00		1.79	0.64	1.00		1.00		1.00	
		(-)		(-)		(0.15–20.77)		(-)		(-)		(-)	
Annual household income													
(2013, Unit: JPY10,000)	<300	ref.		ref.		ref.		ref.		ref.		ref.	
	300-599	1.12	0.85	1.24	0.74	0.73	0.54	0.67	0.51	0.78	0.65	1.04	0.94
		(0.37–3.36)		(0.35–4.41)		(0.27–2.00)		(0.20–2.22)		(0.26–2.31)		(0.33–3.32)	
	600+	0.67	0.54	0.77	0.72	0.58	0.36	0.53	0.36	0.45	0.24	0.56	0.41
		(0.18–2.44)		(0.18- 3.26)		(0.18–1.86)		(0.14–2.06)		(0.12–1.67)		(0.14–2.25)	
	Missing	1.38	0.56	1.66	0.43	1.11	0.84	1.16	0.80	1.03	0.95	1.21	0.73
		(0.46–4.13)		(0.48-5.81)		(0.42–2.93)		(0.37–3.61)		(0.36–2.93)		(0.41–3.61)	

Number of participants for bivariate analysis: a = 214, b = 217, c = 216; otherwise it is 219.

PTSD, post-traumatic stress disorder; OR, odds rario; AOR, adjusted odds ratio; CI, confidence interval.

(−): 95% CI unable to calculate.

Bold signifies p < 0.05.

**Table 4** shows the cross-sectional relationship between parental PTSD symptoms and children’s behavioral problems in 2014 (follow-up). While age, sex, and number of traumatic experiences of the children, age of the parents and annual household income showed no significant association with any type of children’s behavioral problems, parental PTSD symptoms showed a strong positive association with all types of children’s behavioral problems in multivariate analyses (total: AOR = 3.03, internalizing: AOR = 3.30, externalizing: AOR = 5.34). In contrast, having two or more siblings was strongly negatively associated with all types of children’s behavioral problems (total: AOR = 0.17, internalizing: AOR = 0.22, externalizing: AOR = 0.14) and an educational attainment level among parents of “more than high school” was also negatively associated with total and external children’s behavioral problems (AOR = 0.30 and 0.13, respectively) in multivariate logistic regression analyses.

**Table 4:** Association between parental PTSD symptoms and children’s behavioral problem in 2014.

		Total behavior problems				Internalizing behavior problems				Externalizing behavior problems			
		Bivariate		Multivariate (n = 159)		Bivariate		Multivariate (n = 159)		Bivariate		Multivariate (n = 159)	
		OR	p	AOR	p	OR	p	AOR	p	OR	p	AOR	p
		(95%CI)		(95%CI)		(95%CI)		(95%CI)		(95%CI)		(95%CI)	
Parental PTSD Symptoms													
	Non clinical	ref.		ref.		ref.		ref.		ref.		ref.	
	Clinical	2.35	0.06	**3.03**	**0.04**	**3.02**	**0.01**	**3.30**	**0.02**	**3.58**	**0.01**	**5.34**	** <0.01**
		(0.96–5.75)		**(1.06–8.67)**		**(1.27–7.16)**		** (1.25–8.70)**		** (1.38–9.30)**		**(1.64–17.40)**	
Age of child (years)													
		1.17	0.33	1.23	0.25	1.04	0.78	1.04	0.82	0.99	0.96	0.95	0.77
		(0.86–1.59)		(0.87–1.74)		(0.79–1.37)		(0.74–1.46)		(0.73–1.35)		(0.66–1.36)		
Sex of child													
	Boy	ref.		ref.		ref.		ref.		ref.		ref.	
	Girl	0.71	0.40	0.69	0.42	0.76	0.50	0.78	0.58	1.48	0.39	1.81	0.29
		(0.31–1.59)		(0.29–1.68)		(0.34–1.69)		(0.33–1.85)		(0.61–3.58)		(0.61–5.36)	
Number of siblings		a				a				a			
	0	ref.		ref.		ref.		ref.		ref.		ref.	
	1	0.52	0.16	0.55	0.23	0.72	0.48	0.82	0.70	0.47	0.14	0.52	0.26
		(0.21–1.28)		(0.20, 1.48)		(0.29–1.78)		(0.31–2.22)		(0.17–1.27)		(0.16–1.65)	
	2+	**0.17**	**0.01**	**0.17**	**0.01**	**0.22**	**0.01**	**0.22**	**0.04**	**0.16**	**0.01**	**0.14**	**0.03**
		**(0.05–0.58)**		**(0.04–0.68)**		**(0.06–0.74)**		**(0.05–0.92)**		**(0.04– 0.62)**		**(0.02–0.85)**	
	Missing	1.00		1.00		1.00		1.00		1.00		1.00	
		(-)		(-)		(-)		(-)		(-)		(-)	
Traumatic experiences													
(number)	0	ref.		ref.		ref.		ref.		ref.		ref.	
	1-2	2.88	0.06	1.92	0.29	1.57	0.42	1.05	0.93	3.25	0.10	2.84	0.16
		(0.94–8.81)		(0.57–6.46)		(0.52–4.76)		(0.31–3.62)		(0.81–12.97)		(0.66–12.25)	
	3-4	1.63	0.41	0.79	0.73	0.96	0.95	0.51	0.31	2.52	0.19	1.45	0.63
		(0.52–5.13)		(0.21–3.03)		(0.33–2.85)		(0.14–1.88)		(0.64–9.97)		(0.31–6.77)	
	5+	0.97	0.97	0.42	0.35	0.65	0.52	0.40	0.19	1.70	0.55	0.80	0.83
		(0.20–4.58)		(0.07–2.54)		(0.18–2.38)		(0.10- 1.56)		(0.30–9.49)		(0.11– 5.83)	
Age of parent (years)													
		1.00	0.94	1.01	0.83	1.01	0.81	1.02	0.66	0.98	0.61	1.02	0.72
		(0.94–1.06)		(0.94–1.08)		(0.95–1.07)		(0.95–1.09)		(0.92–1.05)		(0.93–1.12)	
Parental education		b				b				b			
	High school or less	ref.		ref.		ref.		ref.		ref.		ref.	
	Some college+	**0.38**	**0.02**	**0.30**	** <0.01**	0.56	0.16	0.44	0.08	**0.20**	** <0.01**	**0.13**	** <0.001**
	**(0.16–0.87)**		**(0.12–0.73)**		(0.25–1.26)		(0.18– 1.09)		**(0.07–0.58)**		**(0.04–0.39)**	
	Missing	1.00		1.00		1.00		1.00		1.00		1.00	
		(-)		(-)		(-)		(-)		(-)		(-)	
Annual household income					c								
(2013, Unit: JPY10,000)	<300	ref.		ref.		ref.		ref.		ref.		ref.	
	300–599	0.82	0.70	1.40	0.56	0.38	0.05	0.55	0.27	0.80	0.69	1.56	0.49
		(0.30–2.27)		(0.45–4.36)		(0.14–1.01)		(0.19–1.59)		(0.27–2.36)		(0.44– 5.54)	
	600+	0.38	0.13	0.70	0.59	0.42	0.13	0.72	0.59	0.25	0.07	0.50	0.42
		(0.11 - 1.34)		(0.19–2.60)		(0.14–1.27)		(0.22–2.33)		(0.06–1.10)		(0.10–2.63)	
	Missing	0.92	0.89	0.75	0.69	0.63	0.55	0.63	0.55	0.43	0.34	0.47	0.45
		(0.25–3.31)		(0.18–3.10)		(0.14–2.88)		(0.14–2.85)		(0.08–2.42)		(0.07 - 3.30)	

Number of participants for bivariate analysis: a = 162, b = 160, c = 177, otherwise it is 174.

PTSD, post-traumatic stress disorder; OR, odds ratio; AOR, adjusted odds ratio; CI, confidence interval.

Bold signifies p < 0.05.

### Association Between Parental PTSD Symptoms in 2012 (Baseline) and Children’s Behavioral Problems in 2014 (Follow-Up)

**Table 5** describes the association between parental PTSD symptoms in 2012 (baseline) and children’s behavioral problems in 2014 (follow-up) adjusted for children’s behavioral problems in 2012 and parental PTSD symptoms in 2014, as well as other covariates. The results revealed that parental PTSD symptoms in 2012 exhibited a significant association with children’s internalizing behavioral problems in 2014 (AOR = 4.62), while a moderate but not significant association with children’s total behavioral problems was found in 2014 (AOR = 2.19). In contrast, parental PTSD symptoms in 2014 exhibited a strong significant association with children’s externalizing behavioral problems (AOR = 5.20), and a moderate but not significant association with children’s internalizing behavioral problems (AOR = 2.84)

**Table 5:** Association between parental PTSD symptoms in 2012 and children’s behavioral problem in 2014.

		Total behavior problems				Internalizing behavior problems				Externalizing behavior problems			
		Bivariate		Multivariate (n = 159)		Bivariate		Multivariate (n = 149)		Bivariate		Multivariate (n = 159)	
		OR	p	AOR	p	OR	p	AOR	p	OR	p	AOR	p
		(95%CI)		(95%CI)		(95%CI)		(95%CI)		(95%CI)		(95%CI)	
Parental PTSD Symptoms													
2012	Non clinical	ref.		ref.		ref.		ref.		ref.		ref.	
	Clinical	2.26	0.06	2.19	0.19	**3.03**	**0.01**	**4.62**	**0.05**	**2.30**	0.08	0.89	0.88
		(0.97–5.25)		(0.68–7.08)		**(1.32–6.94)**		**(1.03–20.78)**		(0.91–5.77)		(0.21–3.83)	
Parental PTSD Symptoms													
2014	Non clinical	–	–	ref.		–	–	ref.		–	–	ref.	
	Clinical	–	–	1.85	0.32	–	–	2.84	0.15	–	–	**5.20**	**0.02**
				(0.55–6.28)				(0.68–11.93)				**(1.27–21.37)**	
CBCL Behavior problem (Baseline in 2012)						a							
	Non clinical range	ref.		ref.		ref.		ref.		ref.		ref.	
	Clinical range	**11.25**	** <0.001**	**15.25**	** <0.001**	**9.03**	** <0.001**	**24.15**	** < 0.001**	**12.44**	** <0.001**	**21.30**	** <0.001**
		**(4.24–29.87)**		**(4.79–48.61)**		**(3.80–21.47)**		**(5.84–99.83)**		**(4.44–34.88)**		**(5.63–80.64)**	
	Missing	2.19	0.22	4.89	0.08	1.00		1.00		**5.09**	**0.02**	**22.25**	**0.03**
		(0.64–7.53)		(0.82–29.00)		(-)		(-)		**(1.32–19.64)**		**(1.27–388.58)**	
Age of child (years)													
		1.13	0.42	1.14	0.60	1.06	0.70	**0.56**	**0.03**	0.90	0.48	1.14	0.67
		(0.84–1.53)		(0.71–1.81)		(0.80–1.40)		**(0.33–0.95)**		(0.66–1.21)		(0.62–2.11)	
Sex of child													
	Boy	ref.		ref.		ref.		ref.		ref.		ref.	
	Girl	0.71	0.41	0.53	0.18	0.81	0.61	0.74	0.61	1.31	0.54	1.10	0.88
		(0.32–1.59)		(0.21–1.33)		(0.36–1.81)		(0.23–2.37)		(0.55–3.10)		(0.33–3.70)	
Number of siblings		b				b				b			
	0	ref.		ref.		ref.		ref.		ref.		ref.	
	1	0.54	0.18	0.47	0.18	0.70	0.43	0.55	0.36	0.55	0.21	0.63	0.49
		(0.22–1.32)		(0.15–1.41)		(0.28–1.73)		(0.15–1.96)		(0.21–1.42)		(0.17–2.32)	
	2+	**0.17**	** <0.01**	**0.21**	**0.02**	**0.21**	**0.01**	**0.13**	**0.05**	**0.16**	**0.01**	**0.08**	**0.05**
		**(0.05–0.57)**		**(0.06–0.80)**		**(0.06–0.72)**		**(0.02–0.97)**		**(0.04–0.61)**		**(0.01–0.98)**	
	Missing	1.00		1.00		1.00				1.00		1.00	
		(-)		(-)		(-)				(-)		(-)	
Traumatic experiences													
(number)	0	ref.		ref.		ref.		ref.		ref.		ref.	
	1-2	2.47	0.12	1.77	0.48	1.20	0.75	0.82	0.79	2.10	0.25	2.87	0.33
		(0.79–7.69)		(0.36–8.65)		(0.38–3.79)		(0.19–3.49)		(0.59–7.43)		(0.35–23.74)	
	3-4	1.27	0.69	0.88	0.88	0.74	0.60	0.38	0.24	1.45	0.57	1.30	0.83
		(0.39–4.15)		(0.15–5.11)		(0.24–2.27)		(0.07–1.92)		(0.41–5.16)		(0.12–13.56)	
	5+	0.77	0.75	0.15	0.07	0.51	0.33	**0.09**	**0.02**	1.00	1.00	0.72	0.78
		(0.16–3.76)		(0.02–1.19)		(0.14–1.94)		**(0.01–0.64)**		(0.19–5.14)		(0.07–7.47)	
Age of parent (years)													
		1.00	0.99	1.01	0.82	1.01	0.84	1.08	0.12	0.99	0.70	1.01	0.86
		(0.94–1.06)		(0.92–1.12)		(0.95–1.07)		(0.98–1.18)		(0.93–1.05)		(0.90–1.14)	
Parental education		c				c				c			
	High school or less	ref.		ref.		ref.		ref.		ref.		ref.	
	Some college+	**0.42**	**0.03**	**0.29**	**0.02**	0.56	0.16	0.37	0.06	**0.28**	**0.01**	**0.07**	** <0.01**
		**(0.18–0.94)**		**(0.10–0.81)**		(0.25–1.25)		(0.13–1.03)		**(0.11–0.72)**		**(0.01–0.38)**	
		Missing	1.00		1.00		1.00		1.00		1.00		1.00
			(-)		(-)		(-)		(-)		(-)		(-)
Annual household income													
(2013, Unit: JPY10,000)	< 300	ref.		ref.		ref.		ref.		ref.		ref.	
	300-599	0.90	0.84	1.70	0.35	0.41	0.08	0.96	0.95	0.87	0.81	2.14	0.32
		(0.32–2.51)		(0.56–5.18)		(0.16–1.10)		(0.21–4.30)		(0.29–2.60)		(0.48–9.56)	
	600+	0.41	0.17	0.62	0.57	0.46	0.17	0.87	0.88	0.27	0.08	0.66	0.70
		(0.12–1.45)		(0.12–3.22)		(0.15–1.39)		(0.15–5.10)		(0.06–1.19)		(0.08–5.43)	
	Missing	0.92	0.89	0.70	0.71	0.53	0.40	0.50	0.44	0.81	0.77	0.20	0.16
		(0.25–3.31)		(0.11–4.58)		(0.12–2.34)		(0.09–2.87)		(0.19–3.43)		(0.02–1.92)	

*Number of participants for bivariate analysis: a = 154, b = 165, c = 163, otherwise it is 169*

PTSD: Post-traumatic stress disorder, OR = odds ratio, AOR = adjusted odds ratio, CI = confidence interval.

Bold signifies p < 0.05.

## Discussion

This is the first longitudinal studies of the long-term psychological impact of the GEJE on parents and their children. Our results revealed several main findings: 1) parental PTSD symptoms and children’s behavioral problems declined over 2 years, but only slightly; 2) parental PTSD symptoms and children’s behavioral problem scores both showed a strong correlation between two surveys; 3) a significant association between parental PTSD symptoms and children’s behavioral problems in a cross-sectional setting was only evident in 2014 (3 years after the disaster), not in 2012 (baseline); 4) parental PTSD symptoms in 2012 had a strong and independent positive association with internalizing children’s behavioral problems in 2014; and 5) the number of siblings and a higher educational attainment level of parents showed a strong and negative (protective) association with children’s behavioral problems in 2014. These results strongly indicate the presence of a long-lasting effect of the disaster on both parental mental status and children’s behavioral problems, suggesting also some possible protective factors.

The long-term persistence of parental mental problems and the behavioral problems of their offspring found in this study is consistent with studies of other disasters (17, 37, 38). Havenaar et al. (37) reported that being a mother and living in an affected area were significant risk factors for adverse mental outcomes 6.5 years after the Chernobyl disaster. Thordardottir et al. (38) conducted a multivariate analysis to assess the association between disaster-related factors and current symptoms among childhood survivors of an avalanche and reported that parents’ traumatic reactions were related to children’s PTSD even after 16 years in a follow-up survey. Furthermore, in a longitudinal study among survivors of Hurricane Katrina, which affected Southern Louisiana in the United States in 2005 (39), Lai et al. assessed the mental status of mothers at four time points continuously over the 2 years after the disaster. The results revealed that mothers’ mental status was strongly associated with the mental health symptoms (post-traumatic stress, depression, and anxiety) of their offspring, suggesting that initial mental status of mothers after the disaster had a long-lasting influence on the mental health of their children, even after mothers had recovered from depression (39).

In the current study, the association between parental mental problems and children’s behavioral problems was unexpectedly detected only in 2014, but not in 2012. This finding may indicate that parental mental problems exert their effects on children’s behavior with years of lead time, or may suggest that other unmeasured but at least equally potent direct or indirect factors also caused children’s behavioral problems, obscuring the association between parental mental problems and children’s behavioral problems during the early period after the disaster. The latter possibility appears to be more likely, given the high number of reports suggesting the immediate effects of parental problems on children’s behaviors (16, 17, 39, 40). Because only a small set of family factors were investigated in our study, other unmeasured factors, such as the mental status of other family members, forced lifestyle changes for children, or separation from close friends could have also influenced children’s behaviors. Future studies of this topic should take more familial risk factors or other socio-cultural factors into account to better understand children’s behavioral problems after disasters, and the necessary support for resolving these difficulties.

Importantly, the current study revealed a strong association between parental mental health problems in 2012 and children’s behavioral problems in 2014, independent of children’s behavioral problems in 2012 and parental mental problems in 2014. This finding may suggest that parental mental health problems continue to affect children’s behaviors over a long period. Further study is needed to investigate the pathway of this long-term association among the survived children and parents at the region affected by GEJE. Whichever the case, this finding suggests the importance of careful and long-term monitoring of the children of parents who have developed probable PTSD.

Regarding the mechanisms by which parental mental health problems affect the behavioral problems of their offspring, Yagi et al. assessed the possible pathways from the parental mental illness (including PTSD symptoms) to child behavioral problems in a cross-sectional study of parents and their children affected by the GEJE, which revealed that parental mental illness first causes PTSD symptoms among children, subsequently affecting their behavioral problems (41). Although this possibility remains to be confirmed by further evidence, such a pathway could have operated to cause the association between parental PTSD symptoms and children’s behavioral problems among participants in the current study.

The association between parental PTSD symptoms in 2012 and internalizing behavior problem of children in 2014 was statistically significant, even after adjusting the SES which was not true for externalizing behavior problem. This is consistent with previous study investigating the association between disaster exposure and the internalizing problem in children (42). They reported that the quality of the parent-child relationship was negatively associated with internalizing psychopathology of children. The PTSD symptoms of parents might be considered to be a factor which disturbs raising the quality of the parent-child relationship, and the impact may appear in 2 to 3 years later. Our finding suggests that the internalizing behavior problem of the children might have a long-term association with the parental initial mental health problems. On the other hand, the child’s externalizing behavior problem was associated with the parent’s PTSD symptom only in the cross-sectional setting (both in 2012 and 2014), suggesting that externalizing symptoms was more responsive to parents’ current level of stress. The reason why the cross-sectional relationships were getting more robust in 2014 than 2012 could be supported by the developmental theory (43), which refers that the risks of developing the externalizing behavior problem are higher at the middle childhood to adolescence than the infant. Because the age of the targeted children of the current study was started at range 4 to 6 years (i.e., infant), it could be expected that those association is getting stronger along with their development, year by year.

Among the factors included in SES, income is considered as the critical factor which affects the mental health of the family members. Rijlaarsdam et al. (44) indicated that economic disadvantage affected both internalizing and externalizing problems of children, and maternal depressive symptoms and parenting stress play a vital role within its pathway. However, our primary result was not wholly consistent with the previous study, possibly because our research was implemented in disaster areas. That is, income level was not associated with trauma exposure (data not shown), suggesting that in the current area, income may not be confounder of the association. Further study will be needed to clarify the mechanism of the relationship between familial mental health and economic status under disaster settings.

Finally, in addition to examining the potential factors negatively impacting children’s behavioral problems, our study also suggested the presence of potentially protective factors for children’s behavioral problems, including the number of siblings and higher education level of parents. The association between the number of siblings in all categories of children’s behavioral problems tended to be dose-dependent, suggesting that siblings may have a protective effect on children’s behavioral problems. However, the meaning of the association between parental higher education level and children’s behavioral problems in this study is unclear. We tested the possibility of the existence of a difference in the distribution of parental job-status [employed, unemployed (i.e., housewives), and others] according to their education level, as a proxy for the time spent with children at home. However, we found no difference in the proportion of employment status between education groups. One possibility is that parents with higher educational attainment levels have more positive health-related behaviors in terms of seeking external psychosocial support or information to protect their children. In view of the potency of the association with children’s behavioral problems, this issue should be further explored in future studies to improve the services provided to children affected by disasters.

Several limitations of the current study should be considered. First, our participants were not a representative sample of the parents or children affected by the GEJE. Because participation was on a voluntary basis, children with severe behavioral problems or parents with severe mental disorders who were undergoing counseling or psychiatric treatment may have been less likely to participate in this study. Second, only family-related factors were considered in this study, and even within in the family, the PTSD symptoms were measured only among parents. Further studies should incorporate the mental status of other family members as well as the possible influence of people outside the family, such as others in the neighborhood, preschool, or school, including teachers or friends. Third, although effort was made to maximize participation, the sample size of the current study was relatively small, limiting its statistical power. Fourth, there was no available information regarding participants’ mental health status before the earthquake. Therefore, it is difficult to clearly identify whether mental health problems among participants resulted from the impact of the earthquake or not.

Despite these limitations, it can be concluded from the current study that the impacts of a disaster continue to affect mental health of both parents and their offspring over a long period. These possibilities should be carefully considered in mental health services, such as psychological support or counseling for parents and their offspring in areas affected by disasters.

## Ethics Statement

The original GEJE-FC study was approved by the ethics committees at the National Center for Child Health and Development and Tokyo Medical and Dental University. Ethical approval was also sought for this secondary data analysis from the Kyoto University Graduate School and Faculty of Medicine Ethics Committee.

## Author Contributions

MO, KN, HH, HM, JY, and TF conceived, designed, and conducted the GEJE-FC study. YH analyzed the data and wrote the first draft, and TF, MO-K, and MK finalized the manuscript. All authors approved the final version of the manuscript.

## Funding

This study is supported by a grant from the Ministry of Health, Labour and Welfare (H24-jisedai-shitei-007) JSPS KAKENHI Grant-in-Aid for Scientific Research on Innovative Areas 4801 and additionally a grant from Kyoto University’s GSS program (a Leading Graduate School Program of the Ministry of Education, Culture, Sports, Science and Technology).

## Conflict of Interest Statement

The authors declare that the research was conducted in the absence of any commercial or financial relationships that could be construed as a potential conflict of interest.
